# Recycling Fiber-Reinforced Polyamide Waste from the Automotive Industry: Life Cycle Assessment (LCA) of an Advanced Pyrolysis Process to Reclaim Glass Fibers and Valuable Chemicals

**DOI:** 10.3390/ma18071594

**Published:** 2025-04-01

**Authors:** Blanca María Caballero, Alexander Lopez-Urionabarrenechea, Jean Paul Gonzalez-Arcos, Borja Benjamín Perez-Martinez, Esther Acha, Maider Iturrondobeitia, Julen Ibarretxe, Aritz Esnaola, Maider Baskaran

**Affiliations:** 1 Department of Chemical and Environmental Engineering, Faculty of Engineering of Bilbao, University of the Basque Country (UPV/EHU), Plaza Ingeniero Torres Quevedo, 1, 48013 Bilbao, Spainborjabaltasar.perez@ehu.eus (B.B.P.-M.);; 2 Department of Organic and Inorganic Chemistry, Faculty of Science and Technology, University of the Basque Country (UPV/EHU), P.O. Box 644, 48080 Bilbao, Spain; jeanpaul.gonzalez@ehu.eus; 3 Department of Graphical Design and Project Engineering, Faculty of Engineering of Bilbao, University of the Basque Country (UPV/EHU), Plaza Ingeniero Torres Quevedo, 1, 48013 Bilbao, Spain; 4 Department of Applied Physics, Faculty of Engineering in Bilbao, University of the Basque Country (UPV/EHU), Paseo Rafael Moreno “Pitxitxi” 3, 48013 Bilbao, Spain; 5 Polymer and Composites Technology, Mechanical and Industrial Production Department, Faculty of Engineering, Mondragon Unibertsitatea, Campus Orona Ideo, Fundazioa Eraikuntza, Jauregi Bailara, 20120 Hernani, Spain; aesnaola@mondragon.edu (A.E.);

**Keywords:** glass mat-reinforced thermoplastic, automotive plastics, complex plastic waste, polyamide, pyrolysis, recycling, recycled glass fibers, life cycle assessment

## Abstract

The generation of pyrolysis liquids and gases with poor quality is a limiting factor for the development of the recycling process of fiber-reinforced plastic waste. In this article, the life cycle assessment (LCA) of an advanced two-step pyrolysis process to recycle glass fiber-reinforced polyamide waste is presented. First, the solid waste is pyrolyzed by heating up at 3 °C/min to 500 °C in a tank reactor. The generated volatiles are subsequently thermally cracked at 900 °C in a tubular packed bed reactor. The process is able to reclaim the glass fibers similarly to the conventional one reactor pyrolysis, while producing liquids and gases with better properties. The large quantity of oxygenated pyrolysis oils generated in the conventional pyrolysis are cracked into gaseous hydrocarbons, CO, CO_2_ and a minor aqueous liquid. The pyrolysis gases become the main product of the process, presenting an interesting composition of hydrogen (39.9 vol.%), methane (22.5 vol.%), carbon monoxide (19.5 vol.%) and ethylene (10.8 vol.%). The LCA shows that advanced pyrolysis demonstrates better environmental performance than conventional pyrolysis, avoiding fossil resource scarcity and reducing global warming by half and human carcinogenic toxicity by one third.

## 1. Introduction

Fiber-reinforced plastics (FRPs) are progressively moving into the automotive sector. Their fabulous strength-to-weight ratio allows for the construction of light vehicles, which consequently leads to less fuel consumption and lower CO_2_ emissions per driven kilometer. These materials, reserved until recently for racing cars and top-of-the-range vehicles, are now projected to double in revenue by 2032 in the automotive sector [1]. The main stimuli for this growth are the need for new materials to decrease the weight of some structures in future electric vehicles (e.g., the battery enclosure) and the development of faster FRP manufacturing technologies able to work at the high production volumes that characterize the automotive industry [2]. However, the environmental benefits of using these lightweight materials in vehicles are constrained by the challenges they present to be completely recycled at the end of life. While it is quite clear that fibers can be recovered from FRP and be reused in the automotive sector itself [3,4], there are well-founded uncertainties about the recyclability of the plastic part of these materials, commonly a thermoset resin [5,6]. Therefore, the integration of FRP in the automotive sector may pose a problem in meeting the circularity requirements for vehicle design planed by the European Commission. This proposal includes, among many other objectives, a 30 wt.% recycling target for plastics in vehicles and a recycled plastic content of 30 wt.% in manufactured vehicles in 2031 [7]. Consequently, the market is shifting towards thermoplastic FRP [8].

Thermoplastics are increasingly used over thermoset composites due to their design flexibility, ease of processing and recyclability, making them ideal for cost-effective, high-volume production. In the automotive industry, manufacturing technologies such as injection molding and compression molding are extensively employed for producing semi-structural or interior components with complex geometries [9]. Glass mat-reinforced thermoplastic (GMT) is a type of FRP composed of a thermoplastic matrix and a glass fiber framework. GMT is widely used in the automotive sector due to its high mechanical strength, short forming cycles and dimensional stability. The use of GMT in automotive applications offers several advantages. Its high mechanical strength ensures durability and performance under various conditions. The short forming cycles associated with GMT allow for efficient production processes, reducing manufacturing time and costs. Additionally, the dimensional stability of GMT ensures that components maintain their shape and fit precisely within assemblies, enhancing overall vehicle quality [10]. Thermoplastic FRPs should be, in principle, more recyclable due to the possibility of re-melting their plastic matrix or re-shaping them, but there are still significant challenges around the mechanical recycling of these materials [8,11,12,13].

In this scenario, pyrolysis is the FRP recycling technique that has reached the highest technology readiness level (TRL) up to now [14]. In this process, the plastic matrix is thermally decomposed (without feeding air), generating volatile substances that separate into liquids and gases when cooling down. The fibers are not decomposed during the thermal process and can be recovered together with the carbonization solid products derived from the plastic decomposition [15]. Pyrolysis is a versatile technique that can be applied to FRP containing cured thermoset resins, uncured thermoset resins, thermoplastics and other non-plastic materials (wood, paints and metals), which constitutes a competitive advantage over other FRP recycling processes. Additionally, the pyrolysis gases, rich in light hydrocarbons, can be used as energetic source for the process. However, there is an issue not solved for the moment: the lack of applications for pyrolysis liquids. This product, which theoretically could be used in refineries or as petrochemical feedstock, is usually a very complex mixture of oily and aqueous phases polluted with heteroatoms, which restricts its possible applications [16,17].

The authors of this article have studied a two-step pyrolysis process for many years, where the pyrolysis volatiles are subjected to a thermal or thermal–catalytic treatment before cooling down and condensing. The aim is to minimize the pyrolysis liquids by converting their organic content into hydrogen, carbon monoxide and light hydrocarbons in the pyrolysis gases [18,19]. This advanced pyrolysis process has been successfully applied to manufacturing and end-of-life FRP waste composed of cured and uncured epoxy, polyester and phenolic thermoset resins. The results up to now are promising and the preliminary economic analysis suggests that the process could have competitive advantages compared to conventional pyrolysis [17]. However, the process requires the employment of an additional reactor with associated material and energetic consumption, which suggests that its environmental impact could be greater than that of conventional pyrolysis. Therefore, the main objectives of this article are two: (1) To evaluate the performance of the advanced pyrolysis process applied to a thermoplastic FRP waste from the automotive industry, specifically a polyamide-based GMT. (2) To assess the environmental impact of the advanced pyrolysis in comparison to conventional pyrolysis and to landfill through the life cycle assessment (LCA) methodology. The results presented in this article are regarded as innovative, as there is no published research on the performance and LCA-based environmental analysis of the two-step pyrolysis process for residual glass fiber-reinforced polyamide. The industrial implementation of the recycling process studied in this article could break down the barriers to the mass use of fiber-reinforced plastics in the automotive industry, making GMT a viable and environmentally responsible choice for modern automotive manufacturing.

## 2. Materials and Methods

### 2.1. Raw Material

**Figure 1:**
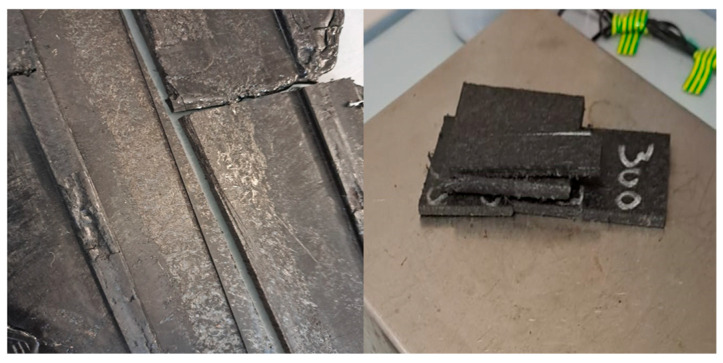
Glass mat-reinforced polyamide-6 composite (**left**, as received; **right**, cuts for experiments).

The sample used in this work was automotive GMT manufacturing waste, based on a glass mat-reinforced polyamide-6 composite. The original GMT was provided by Mitsubishi Chemical Advance, with a glass fiber volume content (υ_f_) of 17% and 1.374 g/cm^3^ material density. The waste was received in monolithic pieces of 10 cm × 30 cm and was cut into samples of 5 cm × 15 cm for the pyrolysis tests (see Figure 1). Furthermore, a small amount of sample was milled down to an approximate particle size of 2 mm for characterization.

### 2.2. Experimental Procedure

**Figure 2:**
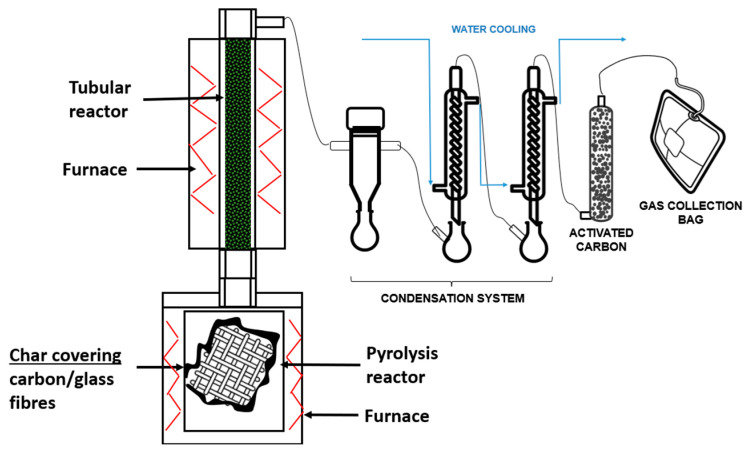
Schematic illustration of the laboratory installation.

Figure 2 shows the experimental setup of the laboratory scale installation used for the conventional pyrolysis and the advanced pyrolysis (with volatiles’ thermal treatment) tests. As can be seen in the scheme, the installation consists of two thermal units connected in series and a condensation and collection system. The first one is formed by a 3 L non-stirred tank reactor heated by an electric furnace, where the pyrolysis is carried out and the solid product generated in the process is deposited. The second unit consists of a fixed bed tubular reactor (2 cm inner diameter and 60 cm long) heated by an electric furnace and connected in series to the first one. In this reactor, the volatiles generated in the pyrolysis reactor are thermally treated, increasing their residence time at high temperatures. Thus, the large pyrolytic molecules are cracked, forming smaller substances, which leads to the minimization of liquids and the maximization of gases. The installation is completed by a condensation system for liquid–gas separation and an activated carbon column for gas cleaning.

In each of the experiments of this work, 100 g of glass fiber-reinforced polyamide were pyrolyzed in the first reactor at 500 °C, using a heating rate of 3 °C min^−^^1^, a dwell time of 30 min and no N_2_ input. In the case that no treatment of volatiles was carried out (conventional pyrolysis), the pyrolysis reactor outlet was connected directly to the condensation system, generating liquids and gases. In the experiments with volatiles’ treatment (advanced pyrolysis), the volatiles produced in the pyrolysis reactor were thermally treated at 900 °C in the tubular reactor (second reactor), containing a solid bed of high alumina refractory brick of 0.5–1 mm particle size. These bricks were a residual material coming from defective bricks and were regenerated after each experiment by combustion at 700 °C in a muffle oven.

Pyrolysis liquids and solid yields were determined by the weight difference in the condensers, pipelines and pyrolysis reactor before and after the experiments, since the gas yield was calculated by the difference to 100.

### 2.3. Analytical Techniques

The initial sample and the pyrolysis products obtained were characterized by employing the following analytical techniques:

– Thermogravimetric analysis

The thermogravimetric profiles of the initial sample were obtained in a Mettler-Toledo (Greifensee, Switzerland) TGA/SDTA851 thermobalance by means of dynamic analysis, heating the sample from 30 to 900 °C under a 10 °C/min heating rate in a 50 mL/min N_2_ atmosphere.

– Proximate analysis

The thermogravimetric balance LECO (St. Joseph, MI, USA) TGA-701 was used for the proximate analysis of the initial sample, following the EN ISO 21660-3:2021 (moisture), EN ISO 22167:2022 (volatile matter) and EN ISO 21656:2021 (ash) standards.

– Elemental analysis (CHNO)

The CHN elemental analysis was determined with a LECO (St. Joseph, MI, USA) TruSpec CHN automatic analyzer according to the EN ISO 21663:2021 standard. The elemental oxygen (O) was analyzed in a Eurovector (Pavia, Italy) Euro EA elemental analyzer.

– Lower Heating Value (LHV)

The automatic calorimeter LECO (St. Joseph, MI, USA) AC-500 was used for the determination of the higher heating value (HHV) of solid and liquid samples, complying with the EN ISO 21654:2022 standard. Then, the LHV was calculated by correcting the HHV with the hydrogen content of the samples. In the case of gases, the LHV was calculated theoretically based on the LHV of the substances composing them.

– Analysis of halogens and sulfur

The elemental analysis of halogens (Cl, Br and F) and sulfur (S) was carried out following the EN 14582:2016 standard. This analysis consists of burning the sample in a calorimeter and absorbing the combustion acid gases (HCl, HBr, HF and SO_2_/SO_3_) in a basic solution. Then, chlorine, bromine, fluorine and sulfur are determined as chloride, bromide, fluoride and sulphate, respectively, by ion chromatography. In this procedure, the LECO AC-500 calorimeter, a NaOH 0.25 M solution and a Dionex (Sunnyvale, CA, USA) ICS 3000 ion chromatograph were used.

– Composition of gases

The composition of gases was quantitatively determined using an Agilent (Santa Clara, CA, USA) 7890A gas chromatograph (GC) with a thermal conductivity detector (TCD) and a flame ionization detector (FID). The GC was a double column chromatograph equipped with a molecular sieve for H_2_, O_2_, N_2_, CO and CH_4_ separation and a column for separation of CO_2_ and hydrocarbons higher than C1. The coordination to split the substances between the molecular sieve and the column is automatized by a valve system in the GC. The GC was calibrated with a standard gas mixture with the following composition: CO_2_ 15 vol.%, H_2_S 5 vol.%, H_2_ 20 vol.%, CO 20 vol.%, CH_4_ 20 vol.%, C_2_H_4_ 5 vol.%, C_2_H_6_ 5 vol.%, C_3_H_6_ 5 vol.%, C_3_H_8_ 3 vol.%, 1-butene 1 vol.% and 1-pentene 1 vol.%.

– Composition of liquids

The composition of liquids was qualitatively determined by gas chromatography (GC, Agilent 6890, Santa Clara, CA, USA) coupled to a mass spectrometer (MS, Agilent 5973, Santa Clara, CA, USA). Such compounds with a match quality provided by the MS search engine lower than 85% were classified as “not identified”. ε-Caprolactam (99%, Sigma-Aldrich, Burlington, MA, USA) was used to calibrate this substance in the GC-MS system in order to get its concentration in wt.%. Four-point calibration was carried out using 1-propanol (99.8%, Supelco, Burlington, MA, USA) as the internal standard.

### 2.4. Life Cycle Assessment (LCA)

The LCA was carried out with the OpenLCA software (version 2.3) coupled with the Ecoinvent v3.10 database, following the methodology of the ISO 14040 standards. The results were categorized into eighteen environmental impact indicators by means of the ReCiPe 2016 Midpoint (H) method [20]. The functional unit (FU) was 1 t of glass fiber=reinforced polyamide. The inventory of the LCA is shown in Table 1, which includes the inputs and outputs of the pyrolysis process in each case, as well as the selected process for landfill. The material and energy inputs were as follows: (1) the energy necessary to heat the reactors using natural gas as fuel, (2) activated carbon used for gas cleaning and (3) water used for cooling the condensers. The energy consumption of the reactors was calculated from a previous work of the authors [19], while the quantity of activated carbon and water was estimated from the data of the laboratory experiments. The output flows representing the pyrolysis products were selected taking into account their characterization results, which will be explained in the “Results and discussion” section. Additionally, the spent activated carbon coming from the gas cleaning was also taken into consideration as a waste output of the pyrolysis processes.

## 3. Results and Discussion

### 3.1. Characterization of the Glass Fiber-Reinforced Polyamide Sample

Table 2 presents the results of the proximate and elemental analysis, as well as the lower heating value (LHV) of the feedstock, including the mean value of at least three equivalent measures that did not differ more than 3 points in wt.% from one to another, and their standard deviation. The analyses were conducted as received (ar) and the results were also calculated as dry and ash free (daf) basis in order to infer the properties of the polyamide matrix. The proximate analysis showed that the sample consisted mainly of volatile matter (69.3 wt.%) and ash (27.0 wt.%), with low moisture and fixed carbon contents (2.3 and 1.4 wt.%, respectively). The low moisture content was expected, since it is typical of plastics, while the ash content was mainly derived from the glass fibers of the sample, together with some inorganic additives that could be part of the GMT formulation, e.g., alumina, kaolin, talcum, calcium carbonate, barium sulfate or titanium dioxide [18]. On the other hand, the daf results showed the nature of the polyamide. It can be seen that the polyamide was constituted almost completely of volatile matter, which indicated the high tendency of the polymer to generate liquids and gases under pyrolysis conditions. On the other hand, the low fixed carbon content meant that the polyamide did not have a tendency to carbonize, which, taking into account that carbonized products remain with the fibers after the pyrolysis process, is a desirable property in terms of reclaiming clean glass fibers through pyrolysis.

Regarding the elemental analysis, the main elements of the sample were carbon (42.9 wt.%), oxygen (8.6 wt.%), nitrogen (8.4 wt.%) and hydrogen (6.4 wt.%). These results were consistent with the nature of polyamide, where the amide functional group is composed of such elements (-CO-NH-). In fact, the results as daf basis corresponded well with those reported in the literature for pure polyamide PA6 [21]. Halogens and sulfur were also analyzed, but the results obtained were below the quantification limit of the equipment, indicating non-existence or trace quantities. Finally, the LHV of the sample presented a typical value for plastics reinforced with similar glass fiber content (≅20 MJ kg^−1^) [22]. Its value as daf basis corresponded well also with that reported in the literature for polyamide [21].

**Figure 3:**
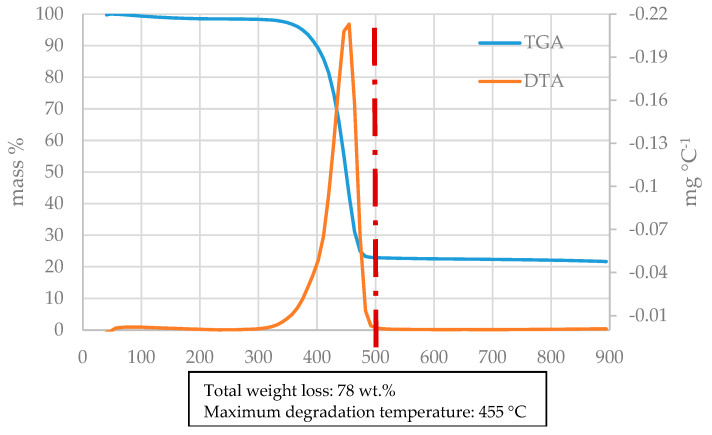
Thermogravimetric behavior of the glass fiber-reinforced polyamide sample.

Figure 3 shows the thermogravimetric behavior of the glass fiber-reinforced polyamide sample. In this figure, a dash line has been added at 500 °C, the temperature of the pyrolysis experiments. The thermogravimetric profile (TGA) allows observing of the weight loss of the sample when it is heated from room temperature up to 900 °C, while the first derivative of the mass loss (DTA) allows determining of the temperature at which the highest rates of weight loss occurs. This analysis can help to decide the most appropriate pyrolysis temperature for the sample. It can be observed that the FRP sample decomposed in a single step in the interval 320–500 °C, presenting the maximum degradation rate at 455 °C. Similar thermogravimetric behavior has been reported by other authors with glass fiber-reinforced polyamide6 [23], pure polyamide PA6 [24] and nylon (polyamide) [25]. In this case, it is clear that 500 °C is a high enough temperature in order to obtain the complete degradation of the polyamide, since no significant weight loss was observed from 500 °C to 900 °C. The remaining weight of the sample, which should approximately correspond to the sum of glass fiber and fixed carbon content, was lower than the ash content of the sample (22 wt.% vs. 27 wt.%). This was probably due to a loss of fiber as a consequence of the sample milling process, since glass fibers have the tendency to agglomerate, generating fiber-enriched zones and resin-enriched zones in the milled sample. This effect can be more pronounce when low sample quantities are employed, as is the case in TGA analysis.

### 3.2. Pyrolysis Yields

Table 3 shows the pyrolysis yields obtained for the glass fiber-reinforced polyamide sample studied, without (conventional) and with (advanced) thermal treatment of volatiles. The results shown in the table are the average and standard deviation values of two experiments for each configuration. It can be observed that in the conventional pyrolysis process, the liquid fraction was the main product (59.8 wt.%), followed by the solid fraction (34.8 wt.%) and a small amount of gases (5.4 wt.%). The sum of the pyrolysis liquid and gas yields (65.2 wt.%) was lower than the sum of volatile matter and moisture contents of the original sample (71.2 wt.%). This result indicated that some of the volatile matter of the sample remained in the pyrolysis solid after the experiment, which can be explained by the difference between the temperature used in the volatile matter analysis (950 °C) and the temperature used in pyrolysis (500 °C). The same fact explained that the solid yield was higher than the ash plus fixed carbon contents of the sample (28.4 wt.%).

In the advanced pyrolysis experiments, a significant decrease in the yield of liquids was observed (from 59.8 wt.% to 21.1 wt.%), and consequently an increase in gas yields, which became the main fraction (44.0 wt.%). This result was expected due to the experimental configuration used in advanced pyrolysis experiments, where the pyrolysis volatiles passed through the tubular reactor at 900 °C, increasing their residence time at high temperatures, which promotes the cracking of the pyrolysis volatiles into small substances. This behavior has been repeatedly observed by the authors in previous research carried out with many types of fiber-reinforced plastic waste [18,19], as well as by other authors using a two-reactor configuration [26].

With respect to the pyrolysis solids, the solid yields did not show significant changes (≅35 wt.%). This was also expected, since the operating conditions of the pyrolysis reactor were the same in both types of experiments.

### 3.3. Pyrolysis Gases

**Figure 4:**
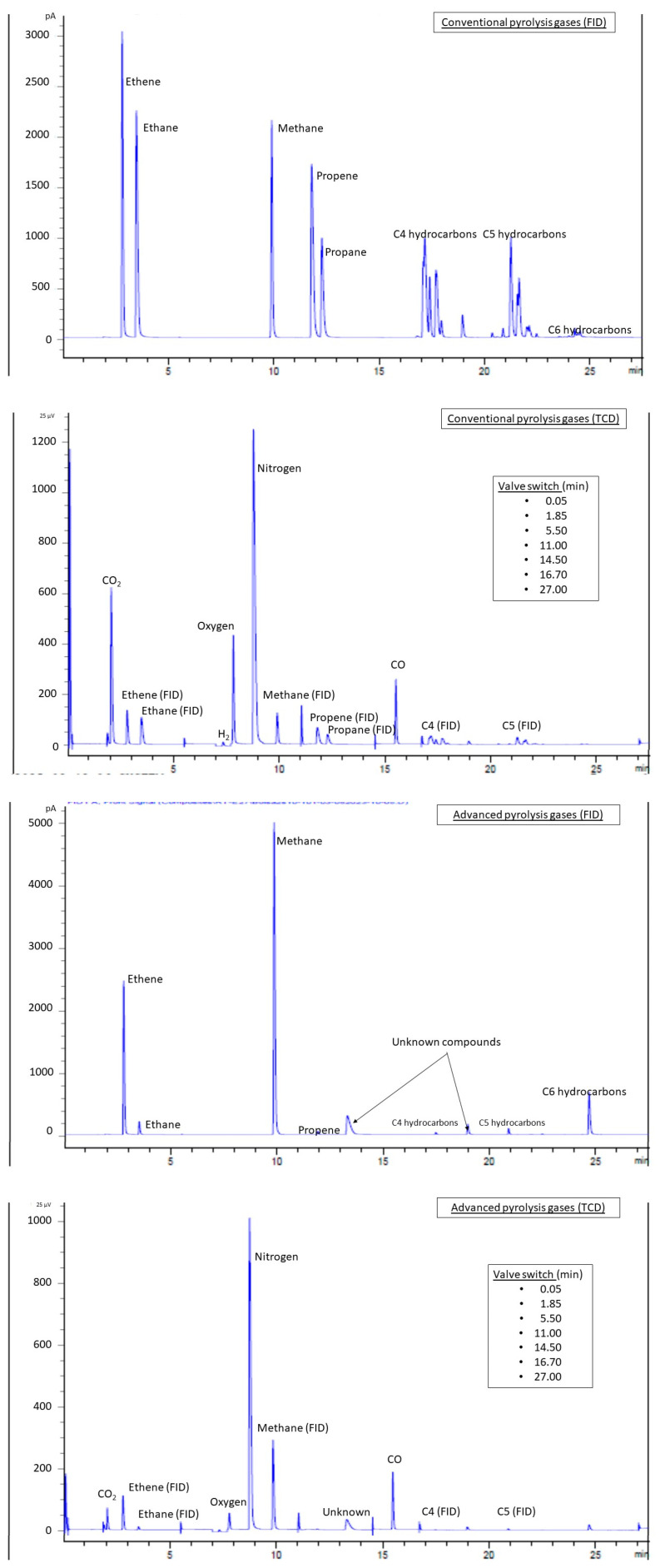
GC chromatograms of pyrolysis gases.

The composition (vol.% and wt.%) and LHV of the pyrolysis gases obtained at 500 °C, without and with volatiles’ thermal treatment at 900 °C, are presented in Table 4. These values are given as dry basis and free of nitrogen, oxygen and other substances that cannot be identified in the GC configuration presented in Section 2.3. The chromatograms generated with the FID and TCD detectors of the GC analysis of the gases are shown in Figure 4.

Table 4 shows that the composition in vol.% of the gases obtained in the conventional pyrolysis experiments was dominated by hydrogen (47.4 vol.%), followed by CO_2_ (18.8 vol.%) and CO (8.4 vol.%), together with hydrocarbons in the range of 2–7 vol.%. The high percentage of hydrogen could suggest the possibility of separating it to produce pure hydrogen. However, the most mature technology for hydrogen separation, the pressure swing adsorption (PSA), is usually employed with feeding gases containing 55 vol.% or higher percentages of hydrogen [27]. Although the percentage of hydrogen of these pyrolysis gases was close to 55 vol.% and there are works that demonstrate that PSA can be used for feeding gases containing 40 vol.% of H_2_ [28], it must be remembered that the gas yield of this experiment was low (5.4 wt.%, Table 3) and this operation would probably not be economically competitive. In fact, the third column of Table 4 shows that the total quantity of hydrogen produced in conventional pyrolysis would be 2.5 g/kg waste, which means a hydrogen yield of approximately 8%. Therefore, these gases could be assimilated to a kind of low quality syngas or may be used to blend with refinery off-gases.

On the other hand, the gases produced in the advanced pyrolysis showed a composition also dominated by hydrogen (39.9 vol.%), methane (22.5 vol.%), carbon monoxide (19.5 vol.%) and ethylene (10.8 vol.%), lowering the concentration of CO_2_ down to 5.6 vol.%. Additionally, it has to be borne in mind that the yield of gases in this experiment was higher than that of the experiment without thermal treatment (44.0 vs. 5.4 wt.%). These gases could find better applications than those of the gases of the conventional pyrolysis due to the following reasons. (1) The concentration of hydrogen is high enough to be separated as pure hydrogen (≈40 vol.%), with a gas production rate of 0.44 kg/kg waste, a H_2_ production rate of 22 g/kg waste and a H_2_ yield of almost 70%. (2) This gas could be a good quality syngas due to the better composition in terms of CO and CH_4_. (3) The proportion of ethylene could open the possibility to blend the gas with the steam cracker off-gases to be used as polyethylene feedstock, which would be the most circular alternative in terms of plastic-to-plastic (P2P) [29].

In order to be able to discuss the formation or consumption tendency of each substance between the two experiments, the total quantity of each gaseous substance was calculated in both cases, without and with volatiles’ treatment. These results showed a specially high formation tendency for CO (×25), CH_4_ (×35) and C_2_H_4_ (×25), and, to a lesser extent, for H_2_ (×9) and CO_2_ (×3), when thermal treatment was used. The high production of light gases is the direct consequence of the high temperature thermal cracking that took place in the tubular reactor. In this reactor, all the pyrolysis volatiles (gases and vapors) suffered a severe thermal cracking that led to the high production of small gaseous molecules. This behavior has been observed before by the authors in their experience working with this kind of waste and plant configuration [18,19]. Concerning the LHV, it can be seen that both gases presented quite similar values, comparable to those of other gaseous fuels such as synthesis gas (10–20 MJ Nm^−3^) and landfill biogas (20 MJ Nm^−3^).

The FID chromatogram of the gas derived from the conventional pyrolysis, given in Figure 4, showed a good definition among C1–C3 hydrocarbons, while the high quantity of substances in the C4 and C5 carbon atom number range led to the typical “dinosaur-like” compilation of non-resolved peaks of complex mixtures. The TCD chromatogram seems to be more complicated by the fact that TCD is more sensitive to valve change and that hydrocarbons also generate peaks in this detector. However, the only signals obtained from TCD were those of CO, CO_2_ and H_2_, the last one with a change of polarity to avoid an inverted peak with respect to the conductivity of helium, used as the carrier gas. In agreement with the gas composition shown in Table 4, the FID chromatogram of the advanced pyrolysis gas clearly showed a more simplified mixture dominated by methane and ethane, while the TCD presented a lower peak for CO_2_.

### 3.4. Pyrolysis Liquids

Table 5 shows the results of the GC-MS analysis, the elemental analysis and the LHV of the pyrolysis liquids, both in conventional and advanced pyrolysis. Regarding the composition of the liquids without volatiles’ treatment, the GC-MS analysis showed a qualitative composition dominated by caprolactam (90.7 area %). In view of such a high area percentage, calibration and quantitative determination of caprolactam in the liquids was carried out, resulting in 43.9 wt.%. This big difference between the GC-MS results in area % and wt.% are typical of some substances when analyzing complex pyrolysis liquids, as the authors demonstrated in a previous paper [30]. Therefore, it should be remembered that equating MS area % to wt.% for some substances present in pyrolysis liquids can result in large errors and a calibration procedure is always needed when the concentration of a single substance is sought. In any case, caprolactam was clearly the main substance in the pyrolysis liquids. The tendency of polyamide to depolymerize into caprolactam by thermal decomposition is a well-known fact in the literature since several years ago [21,23,24,31,32,33]. De Rezende Locatel et al. have recently reported that caprolactam could be formed through two reaction pathways [34]. (1) The cyclization of hexene-5-amide after cleavage of C-N bonds and cis-elimination, and (2) the cyclization of aminocaproic acid after hydrolysis of two amide linkages.

The elemental analysis of the pyrolysis liquid without thermal treatment showed a composition led by carbon (57.3 wt.%), followed by similar proportions of hydrogen, nitrogen and oxygen (≈10 wt.%). These results corresponded quite well with the elemental composition of caprolactam (≈64 wt.% C, 10 wt.% H, 12 wt.% N and 14 wt.% O), the main substance in the liquids, with some differences due to the presence of other substances. However, the high percentage of “others” was surprising, since, attending to the elemental analysis of the FRP sample, they could not be halogens or sulfur. The most probable explanation for this result could be the presence of some inorganic element (Ca or B) coming from the polyamide additives in this pyrolysis liquid, which would not have been burned in the CHN analyzer, being accounted for as “other elements” by the difference to 100. Concerning LHV, a quite high value was obtained for this pyrolysis liquid, similar to that of liquid fossil fuels.

The volatiles’ thermal treatment performed in the advanced pyrolysis completely changed the nature of the pyrolysis liquid, with water being the unique substance identified in the GC-MS. The formation of water during the thermal decomposition of polyamide is assumed in the literature when hydrolysis is identified as one possible reaction step [25,31,32,34]. In this case, the appearance of an aqueous phase as a product was one of the consequences of the cracking of the oxygenated organic substances present in the pyrolysis volatiles. The literature reports that the precursors for the formation of water under pyrolysis conditions are oxygenated functional groups, such as -OH and -COO-, which can be present both in the original sample and in the pyrolysis-derived products [35,36]. These kinds of functional groups can be easily formed during the cracking of the pyrolysis volatiles of polyamide. The elemental analysis revealed a composition dominated by oxygen (47.2 wt.%), followed by similar proportions of hydrogen, nitrogen and carbon (≈ 10 wt.%). Again, the percentage attributed to other elements was high (22.0 wt.%), this time probably due to the fact that the actual percentage of oxygen was underestimated, since the O analyzer is normally calibrated up to 22.1 wt.%. The presence of carbon and nitrogen suggested that this pyrolysis liquid was an aqueous liquid containing organic substances in solution. In order to know what kind of substances could be present in the pyrolysis liquid, a GC-MS analysis in a water-free basis was performed. This analysis revealed the predominant presence of nitriles (propenenitrile, acetonitrile, benzonitrile, butanedinitrile and naphthalenecarbonitrile), which have been previously reported as typical products of the pyrolysis of polyamides [37]. This is attributed to the severe thermal cracking of the organic substances that compose the pyrolysis volatiles to give light hydrocarbons, CO/CO_2_, H_2_ and coke. The results shown in this article demonstrate that this process can be also applied to polyamide with similar results, which constitutes the first time this is applied to a thermoplastic-based FRP.

### 3.5. Pyrolysis Solids

**Figure 5:**
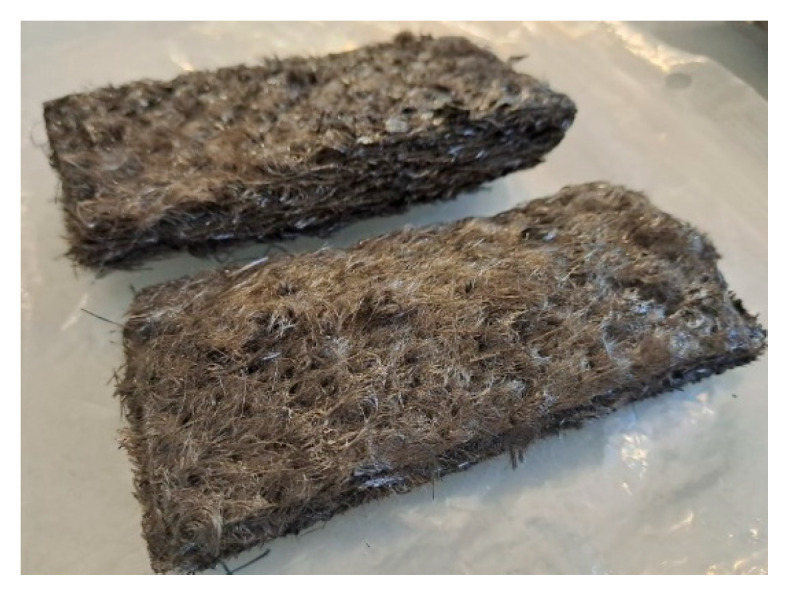
Pyrolysis solid obtained.

Figure 5 shows a sample of the solid product generated in the pyrolysis tests of the glass fiber-reinforced polyamide samples studied in this work. As it can be seen, the pyrolysis solids were a kind of glass fiber covered by char, presenting the typical blackened aspect of the solid product obtained in the pyrolysis of glass fiber-reinforced plastics [38,39]. Table 6 shows the proximate and elemental analyses of the pyrolysis solids obtained both in the tests without and with thermal treatment of the vapors. First, it must be said that the results of both analyses were almost the same for the two samples (without and with thermal treatment), since the pyrolysis operating conditions in the first reactor remained constant.

The presence of carbonaceous substances covering the glass fibers was confirmed by the proximate analysis (Table 6), which showed that the pyrolysis solids still contained around 11 wt.% of organic matter (volatile matter + fixed carbon). As expected, the ash value was the highest one, since the glass fiber content is accounted as ash in the proximate analysis. These results were confirmed by the elemental analysis, where approximately 9 wt.% of carbon was found, together with trace quantities of hydrogen and nitrogen. In this case, the “others” probably corresponded mostly to oxygen. The results of the proximate and elemental analyses of the pyrolysis solids were very similar to those obtained by the authors in previous works concerning pyrolysis of glass FRP [18,19]. These char-covered fibers can be effectively cleaned by a controlled oxidation process in order to obtain recycled glass fibers that could be reused in manufacturing processes [40,41].

### 3.6. Life Cycle Assessment (LCA)

The main goal of the LCA was to assess and compare the environmental impacts of the conventional and the advanced pyrolysis processes, while at the same time comparing these recycling processes with the FRP waste disposal in landfill, the most used treatment route for this type of waste currently. The main difference between the two pyrolysis processes was the energy consumption. As can be seen in Table 1, the energy consumption was estimated to be 3650 kWh/t in the conventional pyrolysis, while it was 7058 kWh/t, almost double, for the advanced pyrolysis. The additional contributions to the energy consumption in the advanced pyrolysis were the use of a second reactor working at high temperatures (900 °C) and the regeneration of the solid bed material of this reactor, which was also thermally done. The energy consumption of the second reactor was lower than that of the first reactor despite working at a higher temperature. There are two main reasons for this: (1) the size of the second reactor is smaller and, consequently, it is normally more efficiently heated, and (2) the vapors entering this second reactor were pre-heated in the first one. Regarding the material consumption of the advanced pyrolysis, the use of the refractory material in the solid bed was not considered to have an environmental impact because it was produced from residual refractory bricks. Additionally, this material can be regenerated and reuse for many cycles without losing its performance.

Both pyrolysis processes were considered to be producers of recycled and reusable glass fibers, while the pyrolysis liquids and gases were assimilated to different products depending on their properties. In the case of the conventional pyrolysis, pyrolysis liquids could be initially considered a source of caprolactam for the production of secondary polyamide. However, more than half of the pyrolysis liquids (56.1 wt.%) were composed of unknown substances, so this pyrolysis liquid should be purified and upgraded through a sequence of different treatment steps before using it as a secondary caprolactam source. Additionally, it must be highlighted that the polymerization quality of caprolactam is very sensitive to impurities and very low quantities of them have been reported as responsible for decreasing its properties [42,43]. Therefore, based on their LHV and the low (or nonexistent) concentration of S and Cl, the selected application for the liquids of the conventional pyrolysis was to be refuse derived fuel (RDF) for cement kilns. Although the concentration of polychlorinated biphenyls (PCBs) and metals should be determined prior to being accepted in cement kilns, this was considered a realistic application for such pyrolysis liquids, taking into account that the concentration of PCB and metals in pyrolysis liquids is low even when high chlorinated and metal-polluted waste is pyrolyzed [29]. Additionally, the FRP waste used in this work was not an end-of-life waste, which is normally more polluted [44]. Concerning the gases of the conventional pyrolysis, they were assimilated to low-quality refinery gases (waste refinery gas) due to the high percentage of CO_2_. In the case of the advanced pyrolysis, the liquids were a kind of industrial wastewater polluted with organic substances. Therefore, an “average” wastewater was selected as the output flow in the LCA software (OpenLCA version 2.3) to represent this liquid fraction. On the other hand, the pyrolysis gases presented a better composition than that of the pyrolysis gases of the conventional pyrolysis, so they were assimilated to refinery gas, a gas to be used in general petroleum refinery operations.

**Figure 6:**
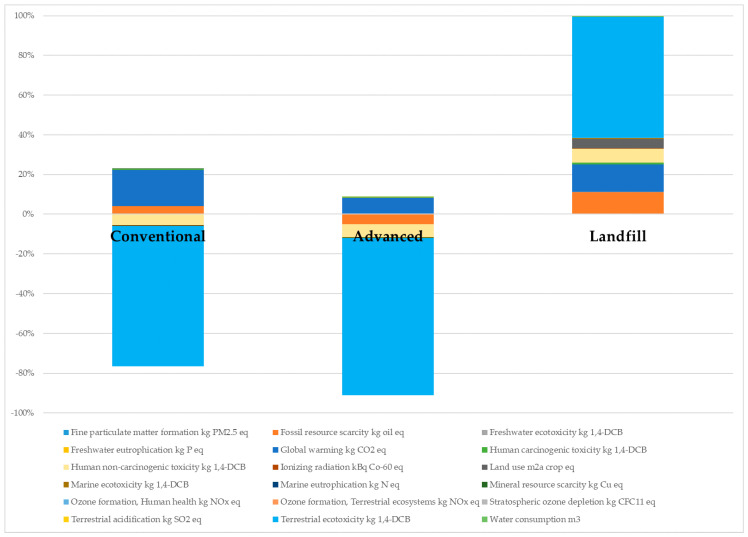
Contribution of each impact category to the total environmental impact of conventional pyrolysis, advanced pyrolysis and landfill.

**Figure 7:**
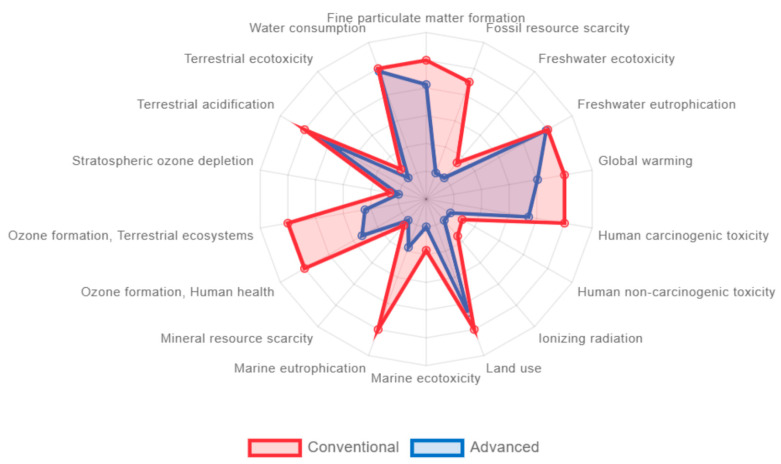
LCA comparison between conventional pyrolysis and advanced pyrolysis.

The results of the LCA are shown in Table 7 and Figure 6 and Figure 7. Table 7 shows the values of all the considered impact categories for the two pyrolysis processes and the landfill. As it can be seen, negative and positive values were obtained, those of landfill being the highest ones by far, various orders of magnitude higher than those of the pyrolysis processes in the majority of the categories. This indicated the worst treatment option for this kind of waste from an environmental point of view. However, Yousef et al. reported that conventional pyrolysis presented a higher environmental impact than landfill in the categories of terrestrial acidification, ozone formation (for both terrestrial ecosystems and human health) and fine particulate matter formation [45]. The key question explaining these differences is if pyrolysis is considered in LCA to be a recycling process where products are obtained. This was demonstrated by Pillain et al., who showed that the environmental impacts of various recycling techniques for FRP waste were higher compared to landfill and incineration when recycled products were not considered [46]. As can be seen in the inventory of the present study (Table 1), recycled glass fibers were considered to be obtained as a product in both pyrolysis processes and, in the case of the advanced pyrolysis, refinery gas was also considered an output.

When recycled products are considered in the LCA, the environmental impacts give negative results in calculation due to the avoided impacts derived from the substitution of primary products and materials by the recycled ones. This is the reason why some values in Table 7 were negative. Therefore, it can be said that both pyrolysis processes avoided impacts in the categories of freshwater ecotoxicity, human non-carcinogenic toxicity, ionizing radiation, marine ecotoxicity, mineral resource scarcity, stratospheric ozone depletion and terrestrial ecotoxicity. Additionally, advanced pyrolysis also avoided impacts in fossil resource scarcity, marine eutrophication and ozone formation (for both terrestrial ecosystems and human health) categories.

The contribution in percentage of the different impact categories is shown in Figure 6. The category with the highest impact in the case under study was terrestrial ecotoxicity. This was responsible for more than 60% of the environmental impact generated by landfill, as well as the main impact avoided by the pyrolysis processes. Figure 6 also shows that 20% of the total environmental impact of the conventional pyrolysis was a real impact affecting the environment, global warming being the highest impact of this process. On the other hand, almost 80% of the impacts were avoided with this treatment compared to landfill. Concerning global warming, Tapper et al. reported higher greenhouse gas emissions for fluidized bed pyrolysis of carbon fiber-reinforced polymers compared to landfill [47], while other studies, and that of this article, showed lower global warming potential for pyrolysis process [45,48]. The reason for such differences could lie on the fact that LCA results are dependent on the quality and reliability of the life cycle inventory, leading to different conclusions for the same processes. The definition of the inventory for the initial reinforced plastic sample itself can have a significant influence, as has been recently demonstrated by Moutik et al. [49]. In the case of the advanced pyrolysis, approximately 90% of the impacts were avoided, global warming again being the highest impact affecting the environment. However, this impact was reduced by half compared to the conventional pyrolysis (1104 vs. 566.8 kg CO_2_ eq).

The comparison of the LCA between the two pyrolysis processes is represented in Figure 7. It can be seen that the advanced pyrolysis showed better environmental performance than conventional pyrolysis in all the impact categories, in some of them with a remarkably better result. This is the case for ozone formation, marine eutrophication and, especially, fossil resource scarcity. The better performance in this last category was probably derived from the fact that the advanced pyrolysis produced as a main product a gas fraction usable as refinery gas, substituting the actual refinery gas coming from fossil resources. Apart from the abovementioned categories, it is also worth noting the reduction of impact in two important categories, namely global warming (by half) and human carcinogenic toxicity (to one-third). These results demonstrate the environmental benefits of using two-stage advanced pyrolysis for the recycling of FRP waste.

## 4. Conclusions

The main conclusions derived from this study are two: (1) the two-stage advanced pyrolysis process can be applied to a residual glass fiber polyamide mat and (2) this process shows a better environmental performance than the conventional pyrolysis that is usually applied to FRP waste. On the one hand, the “difficult-to-use” pyrolysis liquids are converted into wastewater and useful gases through the advanced pyrolysis process, while the glass fibers can be reclaimed and reused, as in conventional pyrolysis. On the other hand, the life cycle assessment shows that pyrolysis processes of glass fiber-reinforced polyamide are environmentally better than the common management option for FRP waste, the landfill. Additionally, when comparing the impacts of the conventional and advanced pyrolysis processes, it can be seen that advanced pyrolysis presents better environmental performance than conventional pyrolysis in all impact categories, among them global warming, human carcinogenic toxicity and, especially, fossil resource scarcity. The outstanding result in this last category is attributed to the generation of large quantities of a refinery gas-like gas fraction by means of the advanced pyrolysis process. The good environmental performance of this process is a new step forward for the consolidation of pyrolysis as a recycling technique for fiber-reinforced plastic waste. Further industrial development could stimulate the use of fiber-reinforced plastics in the automotive industry, since the recyclability of such materials aligns with the growing emphasis on sustainability in this sector.

## Figures and Tables

**Table 1:** Inventory of the LCA study.

Process	Input (I)/Output (O)	Flow	Amount	Unit	Avoided Waste	Provider
Conventional pyrolysis	I	Waste glass fiber PA	1	t		
		Natural gas, burned in gas turbine	3650	kWh	-	Natural gas, burned in gas turbine | natural gas, burned in gas turbine | cutoff, U—ES
		Activated carbon, granular	0.2	t	-	Activated carbon production, granular from hard coal | activated carbon, granular | cutoff, U—RoW
		Tap water	20	t	-	Tap water production, conventional treatment | tap water | cutoff, U—RoW
	O	Waste refinery gas	1458	MJ	-	Treatment of waste refinery gas, burned in flare | waste refinery gas | cutoff, U—GLO
		Waste mineral oil	0.598	t	-	Clinker production | waste mineral oil | cutoff, U—Europe without Switzerland
		Glass fiber	0.27	t	TRUE	Glass fiber production | glass fiber | cutoff, U—RoW
		Spent activated carbon	0.2	t	-	Spent activated carbon, granular, recycled content cut-off | spent activated carbon, granular | cutoff, U—GLO
Advanced pyrolysis	I	Waste glass fiber PA	1	t		
		Natural gas, burned in gas turbine	7058	kWh	-	Natural gas, burned in gas turbine | natural gas, burned in gas turbine | cutoff, U—ES
		Activated carbon, granular	0.2	t	-	Activated carbon production, granular from hard coal | activated carbon, granular | cutoff, U—RoW
		Tap water	20	t	-	Tap water production, conventional treatment | tap water | cutoff, U—RoW
	O	Refinery gas	0.44	t	TRUE	Refinery gas production, petroleum refinery operation | refinery gas | cutoff, U—RoW
		Wastewater, average	0.211	t	-	Treatment of wastewater, average, wastewater treatment | wastewater, average | cutoff, U—Europe without Switzerland
		Glass fiber	0.27	t	TRUE	Glass fiber production | glass fiber | cutoff, U—RoW
		Spent activated carbon	0.2	t	-	Spent activated carbon, granular, recycled content cut-off | spent activated carbon, granular | cutoff, U—GLO
Landfill						Process-specific burdens, residual material landfill | process-specific burdens, residual material landfill | cutoff, U

**Table 2:** Chemical analysis of the glass fiber-reinforced polyamide sample.

Proximate Analysis (wt.%)	As Received (ar)	Dry and Ash Free Basis (daf)
Moisture	2.3 ± 0.01	-
Volatile matter	69.3 ± 1.0	98.0 ± 1.0
Ash	27.0 ± 1.3	-
Fixed carbon ^1^	1.4	2.0
**Elemental Analysis (wt.%)**	**As Received (ar)**	**Dry and Ash Free Basis (daf)**
Carbon	42.9 ± 2.8	60.7 ± 2.8
Hydrogen	6.4 ± 0.4	9.0 ± 0.4
Nitrogen	8.4 ± 0.1	11.9 ± 0.1
Oxygen	8.6 ± 1.8	12.2 ± 1.8
Fluorine, chlorine, bromine	u.q.l. ^2^	u.q.l. ^2^
Sulfur	u.q.l. ^2^	u.q.l. ^2^
Others ^1^	4.4	6.2
**LHV (MJ kg^−1^)**	**20.6 ± 0.34**	**29.1 ± 0.34**

^1^ calculated as 100-moisture-ash-C-H-N-O, ^2^ under quantification level. The three different analyses are indicated in bold in the table.

**Table 3:** Pyrolysis yields (wt.%).

	Conventional Pyrolysis	Advanced Pyrolysis
Solid	34.8 ± 3.6	34.9 ± 2.4
Liquid	59.8 ± 3.1	21.1 ± 0.3
Gas ^1^	5.4 ± 0.4	44.0 ± 0.1

^1^ By difference.

**Table 4:** Composition and LHV of the pyrolysis gases obtained without and with volatiles’ thermal treatment.

	Conventional Pyrolysis			Advanced Pyrolysis		
	vol.%	wt.%	Total Quantity (g) ^1^	vol.%	wt.%	Total Quantity (g) ^1^
H_2_	47.4	4.6	0.25	39.9	5.0	2.20
CO	8.4	11.4	0.62	19.5	33.8	14.87
CO_2_	18.8	40.2	2.17	5.6	15.2	6.69
CH_4_	6.6	5.1	0.28	22.5	22.8	10.03
C_2_H_4_	4.5	6.1	0.33	10.8	18.7	8.23
C_2_H_6_	4.5	6.7	0.36	0.7	1.4	0.62
C_3_H_6_	2.9	6.0	0.32	0.2	0.5	0.22
C_3_H_8_	1.6	3.4	0.18	0.3	0.8	0.35
C_4_	3.3	9.5	0.51	0.2	0.7	0.31
C_5_	2.0	6.9	0.37	0.2	1.0	0.44
LHV	20.9 MJ/Nm^3^	27.0 MJ/kg		19.1 MJ/Nm^3^	31.7 MJ/kg	
H_2_ yield ^2^	7.8%			68.8%		

^1^ Gas yield * wt.% of each substance; ^2^ [(Total quantity of H_2_) × 2/H in the waste] × 100.

**Table 5:** Elemental analysis (wt.%) and LHV (MJ kg^−1^) of the liquids obtained without and with volatiles’ thermal treatment.

	Conventional Pyrolysis	Advanced Pyrolysis
	GC-MS analysis (area %)	
Caprolactam	90.7 (43.9 wt.%)	n.d. ^2^
Water	n.d. ^2^	100
Other identified	1.3	n.d. ^2^
Not identified	8.0	n.d. ^2^
	Elemental analysis	
Carbon	57.3 ± 0.1	9.9 ± 9.1
Hydrogen	9.6 ± 0.4	9.8 ± 0.1
Nitrogen	10.9 ± 0.1	11.1 ± 0.1
Oxygen	10.9 ± 0.4	47.2 ± 2.5
Others ^1^	11.3	22.0
LHV (MJ kg^−1^)	42.5 ± 4.01	- ^3^

^1^ By difference. ^2^ Not detected. ^3^ No combustible sample.

**Table 6:** Proximate and elemental analysis (wt.%) of the pyrolysis solids without and with volatiles’ treatment.

Proximate Analysis	Conventional Pyrolysis	Advanced Pyrolysis
Moisture	0.4 ± 0.0	0.3 ± 0.2
Volatile matter	4.6 ± 0.2	4.3 ± 0.3
Ash	88.0 ± 0.2	88.8 ± 4.1
Fixed carbon ^1^	7.0	6.5
**Elemental Analysis**	**Conventional Pyrolysis**	**Advanced Pyrolysis**
Carbon	8.9 ± 0.2	8.6 ± 0.0
Hydrogen	0.4 ± 0.0	0.2 ± 0.0
Nitrogen	0.6 ± 0.1	0.6 ± 0.0
Others ^2^	1.7	1.5

^1^ By difference. ^2^ Calculated as 100-moisture-ash-C-H-N.

**Table 7:** Results of the LCA of conventional pyrolysis, advanced pyrolysis and landfill of glass fiber-reinforced polyamide.

Impact Categories	Conventional	Advanced	Landfill
Fine particulate matter formation (kg PM_2.5_ eq)	2.165	1.216	4597
Fossil resource scarcity (kg oil eq)	248.8	−335.3	2.44 × 10^6^
Freshwater ecotoxicity (kg 1,4-DCB)	−5.998	−9.289	6.04 × 10^4^
Freshwater eutrophication (kg P eq)	0.505	0.488	339
Global warming (kg CO_2_ eq)	1104	566.8	3.06 × 10^6^
Human carcinogenic toxicity (kg 1,4-DCB)	21.72	7.638	1.67 × 10^5^
Human non-carcinogenic toxicity (kg 1,4-DCB)	−331.2	−436.1	1.55 × 10^6^
Ionizing radiation (kg Bq Co-60 eq)	−5.735	−9.116	5.89 × 10^4^
Land use (m^2^a crop eq)	9.962	6.743	1.07 × 10^6^
Marine ecotoxicity (kg 1,4-DCB)	−7.992	−13.86	8.87 × 10^4^
Marine eutrophication (kg N eq)	0.022	−0.012	171.8
Mineral resource scarcity (kg Cu eq)	−5.068	−5.744	7520
Ozone formation, Human health (kg NOx eq)	1.394	−0.252	1.42 × 10^4^
Ozone formation, Terrestrial ecosystems (kg NOx eq)	1.383	−0.544	1.54 × 10^4^
Stratospheric ozone depletion (kg CFC11 eq)	−6.1 × 10^−4^	−7.3 × 10^−4^	0.844
Terrestrial acidification (kg SO_2_ eq)	6.289	3.416	1.10 × 10^4^
Terrestrial ecotoxicity (kg 1,4-DCB)	−4303	−5385	1.35 × 10^7^
Water consumption (m^3^)	18.73	17.72	1.41 × 10^5^

## Data Availability

The original contributions presented in this study are included in the article. Further inquiries can be directed to the corresponding author.
